# Report of Two Cases of Hydroa

**Published:** 1883-05

**Authors:** Edwin R. Bennett

**Affiliations:** Cook County Hospital, Chicago


### ARTICLE IV.

REPORT OF TWO CASES OF HYDROA. By EDWIN R. BENNETT, M.D., Cook County Hospital, Chicago.

Prof. Duhring defines hydroa as an acute inflammatory disease, characterized by one or more groups of variously sized vesicopapules or vesicles, arranged in the form of concentric rings, attended as a rule by the display of varied colors. All cases of a disease are not typical therefore. I present the following cases taken from the records of the Cook County Hospital, as presenting some interesting points;

Case 1. J. R.; Swede; æt. 21; occupation, clerk.

No history of any previous sickness, or family taint.

Only veneral lesion, gonorrhœa; all lesions pointing to syphilis wanting; drinks beer daily, moderately.

Admitted to hospital September 8, 1882.

One and one half weeks before admission, without any known cause, patient discovered reddish macules, shortly becoming vesicles and in some instances the vesicles uniting to form bullæ, appearing on the backs of hands, fore-arms, legs and feet, varying in size from pin head, to that of a hickory nut. In from twenty-four to forty-eight hours after the first appearance of the lesions, the contents which before were yellowish serum, would in some instances disappear without further change, while in others the contents become cloudy, soon rupturing and drying into a lightish scab, which would disappear in a few days. In certain instances when the macules were violently ruptured, an inflammatory condition of the base would ensue, with the production of a few drops of pus. It was also noted that while it was developing in one part, it would be undergoing retrograde metamorphosis in another.

Neither did the lesions assume a distinctly circular form nor present the variegated and delicate tints that gave us the German name herpes iris.

Its location and symmetry are to be observed, occurring with remarkable regularity on both hands, forearms, legs and feet, dorsal aspect only in each case. An entire absence of constitutional disturbance was noted, and the only local symptom was slight soreness. Cod liver oil was given internally and carbolized cosmoline locally. In a few weeks the patient was discharged cured.

Case. 2. J. M.; æt. 25; English; lawyer by occupation. Habits temperate. Only venereal accident, gonorrhœa. Admitted October, 6 1882.

Present trouble began August 1, by devolpment of vesicles on back of hands and forearms. There were no bullæ, each vesicle ruptured spontaneously, some disappearing without further change, in others the contents dried in a lightish scab which was quickly exfoliated, leaving a reddened base. Order of appearance; 1. hands, dorsal aspect; 2. forearms, extensor aspect; 3. dorsal surface of feet, and lastly extensor surface of legs.

The disease has not affected his general health in the least, but on account of its distribution the patient imagines it is a severe disease and wants prognosis and treatment. A few days afterward patient was discharged cured. The treatment being entirely local and confined to a dusting powder.

An examination of the history given shows us that the disease in question, is in reality a harmless one, although formidable in appearance to the patient, and possessing characteristics sufficient to distinguish it from other vesicular, or vesiculo-bullous eruptions, the most of which are not harmless complaints. It is to be diagnosed from zoster by the absence of neuralgic pain and burning, and the situation, it not following the distribution of nerves.

From pemphigus, it is distinguished by being confined to certain limited regions and causing no constitutional disturbance.

From eczema, by absence of itching and infiltration of tissues.

It is to be distinguished from all by its harmlessness.

All the medication required is a local dusting powder, its importance attaching itself to the diagnosis to prevent us from treating it for something worse.

